# *Helicobacter Pylori* and Autoimmune Diseases: Involving Multiple Systems

**DOI:** 10.3389/fimmu.2022.833424

**Published:** 2022-02-10

**Authors:** Li Wang, Zheng-Min Cao, Li-Li Zhang, Xin-can Dai, Zhen-ju Liu, Yi-xian Zeng, Xin-Ye Li, Qing-Juan Wu, Wen-liang Lv

**Affiliations:** ^1^Department of Infection, Guang’anmen Hospital, China Academy of Chinese Medical Sciences, Beijing, China; ^2^Department of Proctology, Guang’anmen Hospital, China Academy of Chinese Medical Sciences, Beijing, China; ^3^Department of Cardiovascular, Guang’anmen Hospital, China Academy of Chinese Medical Sciences, Beijing, China

**Keywords:** *Helicobacter pylori*, autoimmune diseases, relationship, pathogenesis, review

## Abstract

The modern Gastroenterology have witnessed an essential stride since *Helicobacter pylori* was first found in the stomach and then its pathogenic effect was discovered. According to the researches conducted during the nearly 40 years, it has been found that this bacterium is associated with a natural history of many upper gastrointestinal diseases. Epidemiological data show an increased incidence of autoimmune disorders with or after infection with specific microorganisms. The researches have revealed that *H. pylori* is a potential trigger of gastric autoimmunity, and it may be associated with other autoimmune diseases, both innate and acquired. This paper reviews the current support or opposition about *H. pylori* as the role of potential triggers of autoimmune diseases, including inflammatory bowel disease, autoimmune thyroiditis, type 1 diabetes mellitus, autoimmune liver diseases, rheumatoid arthritis, idiopathic thrombocytopenic purpura, systemic lupus erythematosus, as well as Sjogren’s syndrome, chronic urticaria and psoriasis, and tried to explain the possible mechanisms.

## 1 Background and Epidemiology

*Helicobacter pylori* has been co-evolving with humans for more than 60,000 years (1), and it was first isolated from stomach biopsy in 1983 by Marshall and Warren (2). *H. pylori* is a bacterium which is spiral-shaped, microoxic and Gram-negative, and over 50% of people worldwide are suffering from its infection (3). The prevalence rate of the disease is positively correlated with age growth and low socioeconomic status. Still, due to the geographical location and the specific patient population, there is a big difference (4). It has been proved that the poor living conditions in childhood is the crucial risk factor for people to get infected by *H. pylori* (5). Certain ethnic minorities and immigrants have higher levels of infection (6).

*H. pylori* is generally transmitted from mouth to mouth and feces to mouth, and contaminated water supplies may also be another source of transmission (7). It is mainly obtained in childhood and is likely to last for a life time of the host (8). Childhood spontaneous remission is relatively common but it’s often associated with diseases treated with antibiotics (9).

Clinically, there are plenty of diseases associated with *H. pylori*, such as peptic ulcer diseases, autoimmune gastritis, gastric cancer, as well as some other illnesses like iron deficiency, vitamin B12 deficiency, and idiopathic thrombocytopenia (10). Currently, the treating methods of *H. pylori* mainly include triple therapy, sequential therapy, bismuth therapy, vonoprazan, probiotics, vaccines, etc. (11) But some current problems such as antibiotic resistance and high recurrence rate still need to be concerned.

## 2 *Helicobacter Pylori* and Immune System

**Figure 1:**
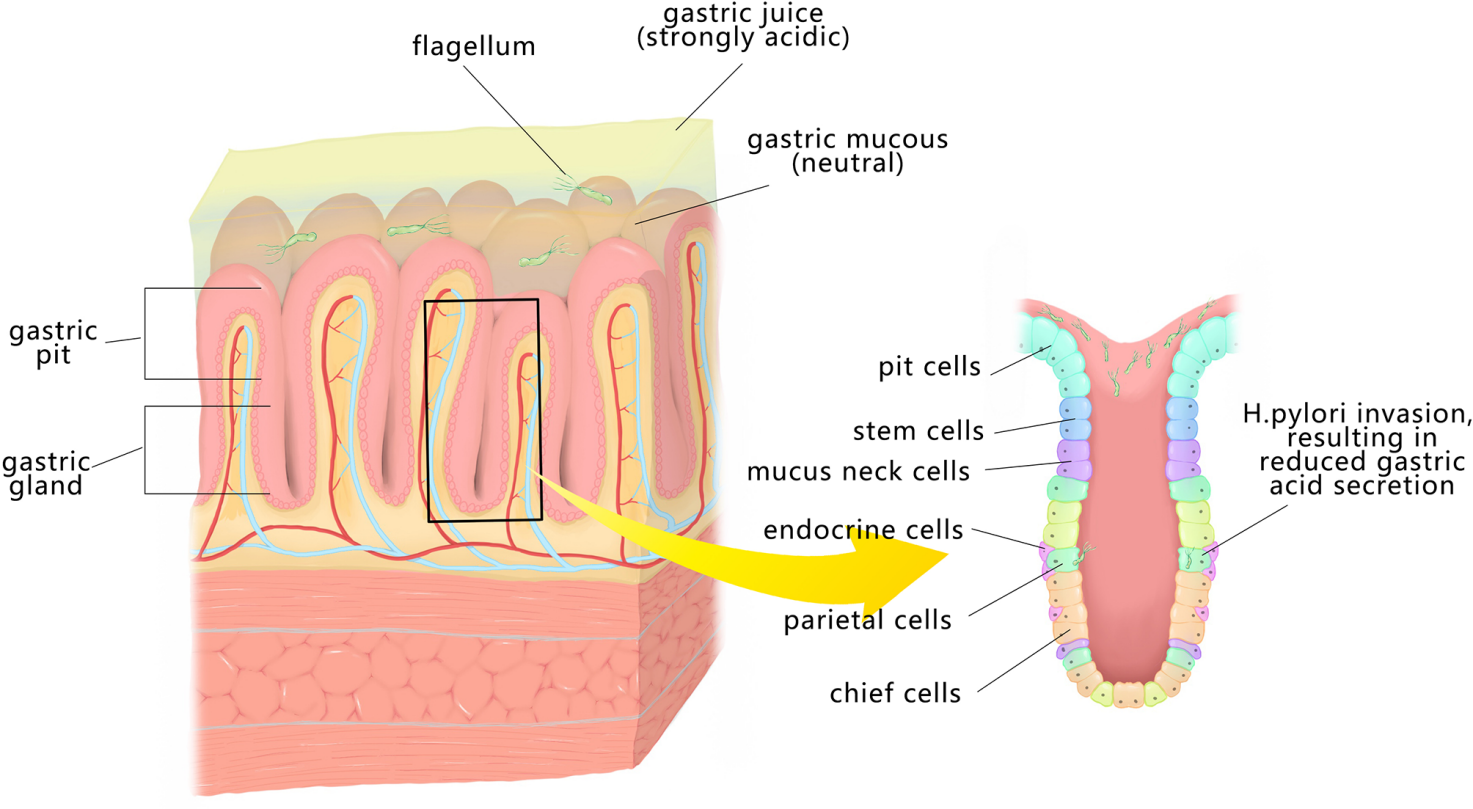
*H. pylori* is spirally shaped and has flagella that enable it to pass through the mucus layer to the surface of GECs. It can produce urea enzyme to decompose urea, and then generate ammonia, so that the pH increases, which is conducive to its customization. It can also invade gastric epithelial cells, secrete toxin factors, encourage inflammation and immune response, and destroy the gastric mucosa.

*H. pylori* should be able to tolerate the gastric acidic within the stomach for survival. Meanwhile, the immune mechanisms of human body may also cause fatal threat to it. In order to avoid being eliminated, it has no choice but to develop a variety of mechanisms. With the flagella, its movement is promoted and it is able to persist in the stomach lining (**Figure 1**). It produces urease, which on the one hand, converts urea to carbon dioxide and ammonia, enabling it to overcome the acidic environment; on the other hand, it changes the viscosity of gastric mucus and promote bacterial movement. Some of the toxins effector/proteins released by *H. pylori* include cytotoxin-associated gene A(CagA), outer membrane vesicles (OMV), outer inflammatory protein (OipA), vacuolating cytotoxin gene A (VacA), high-temperature requirement A (HtrA), outer membrane protein (OMP), neutrophil-activating protein A (NepA), et.al (12). The two virulence factors of *H. pylori* CagA and VacA have been widely researched. CagA is the first *H. pylori* virulence factor related to more severe illnesses and can affect many cellular processes. In addition, VacA has multiple functions, from inducing apoptosis to regulating the immune system (13). Both affect cell shape and affect immune cells, which may be the cause of elevated levels of autoimmune antibodies (13).

After fixing in the gastric epithelium, *H. pylori* activates the immune system of the body, which is manifested in the synthesis of various proinflammatory cytokines by mucosal epithelial cells, and then induces many kinds of immune cells to accumulate in the gastric mucosa after activation. The activated immune cells further secrete a variety of cytokines, promote the effective presentation of *H. pylori* antigen components to T cells and B cells, and produce particular cellular immunity and humoral immunity. The possible immune mechanism will be analyzed from three aspects: immune recognition, innate immune response and adaptive immune response.

Pattern recognition receptors (PRRs) distributed on host gastric epithelial cells (GECs). Neutrophils and antigen presenting cells (APCs) identify the pathogen associated molecular patterns (PAMPs) of *H. pylori*, and then initiate the immune response to *H. pylori* (14). *H. pylori* type-IV secretion system (T4SS) is a needle-like structure protruding from the surface of bacteria, which can pierce the host cells, and transport cytotoxin-associated gene A product (CagA) or other pathogenic factors into host cells. After *H. pylori* infection, firstly, the biological precursor of Lipopolysaccharide (LPS) delivered through T4SS enters the host cytoplasm, activates tumor necrosis factor receptor associated factor (TRAF), etc., and then starts the related response (15). LPS can act as a superantigen, causing extensive lymphocyte activation.

Antigen recognition of innate immunity can be regulated by APCs such as monocytes and dendritic cells. GECs recognize *H. pylori* antigen and secrete chemokines, such as interleukin-8 (IL-8), CXC chemokine ligand, CC chemokine ligand 20, followed by recruitment of neutrophils, eosinophils, monocytes, and macrophages to the site of infection (16).

After *H. pylori* infection, antigen-specific B cells and T cells appear in the host. The dendritic cell located in Peyer’s patches(PPs) recognizes the antigen and activates the primitive T cells in the PPs (17). After T cells are activated, they can cause two responses: Th1 response and Th2 response, mainly CD4+T helper cells aggregate in lamina propria. *H. pylori* specific CD4+T cells could be detected in gastric mucosa of *H. pylori*-infected patients (18).

Different T-cell responses induced by *H. pylori* produce different cytokines. According to the research, the components of *H. pylori*, specifically urease, are able to promote B cells to secrete anti-double-stranded deoxyribonucleic acid(DNA), IgM rheumatoid factor, and anti-phospholipid choline antibodies (19). From *H. pylori* adhesive colonization, immune cells activate, secrete cytokines, and produce specific humoral immunity against *H. pylori*. Certain antigenic components of *H. pylori* have a similar structure to gastric mucosal epithelial tissues, which can lead to the occurrence of cross reactivity through molecular mimicry and/or epitope diffusion mechanisms (20). For instance the β subunit of urease in *H. pylori* is highly homologous to the β subunit of ATPase in gastric parietal cells thus leading to autoimmune reaction (20). Within the *H. pylori*-positive patients’ stomach, cytokines such as interferon-γ (IFN-γ), tumor necrosis factor-α (TNF-α), interleukin-1 (IL-1), etc. are at high levels (21). IFN-γ can make parietal cells as APC targets for cross-reaction epitope recognition, leading to death or apoptotic suicide (22).

In addition, *H. pylori* can evade the host immune response in a variety of ways, so that the innate and specific immunity is not enough to clear the infection and enable bacteria to colonize and form chronic infection. For example, the bacteria can modify antigens that reside on the cell wall, like bacterial endotoxin LPS and flagella, making the two potential antigens comparatively weak (23). In addition, *H. pylori* regulates T cell immune responses through virulence factors (24).

## 3 *Helicobacter Pylori* and Autoimmune Diseases

**Figure 2:**
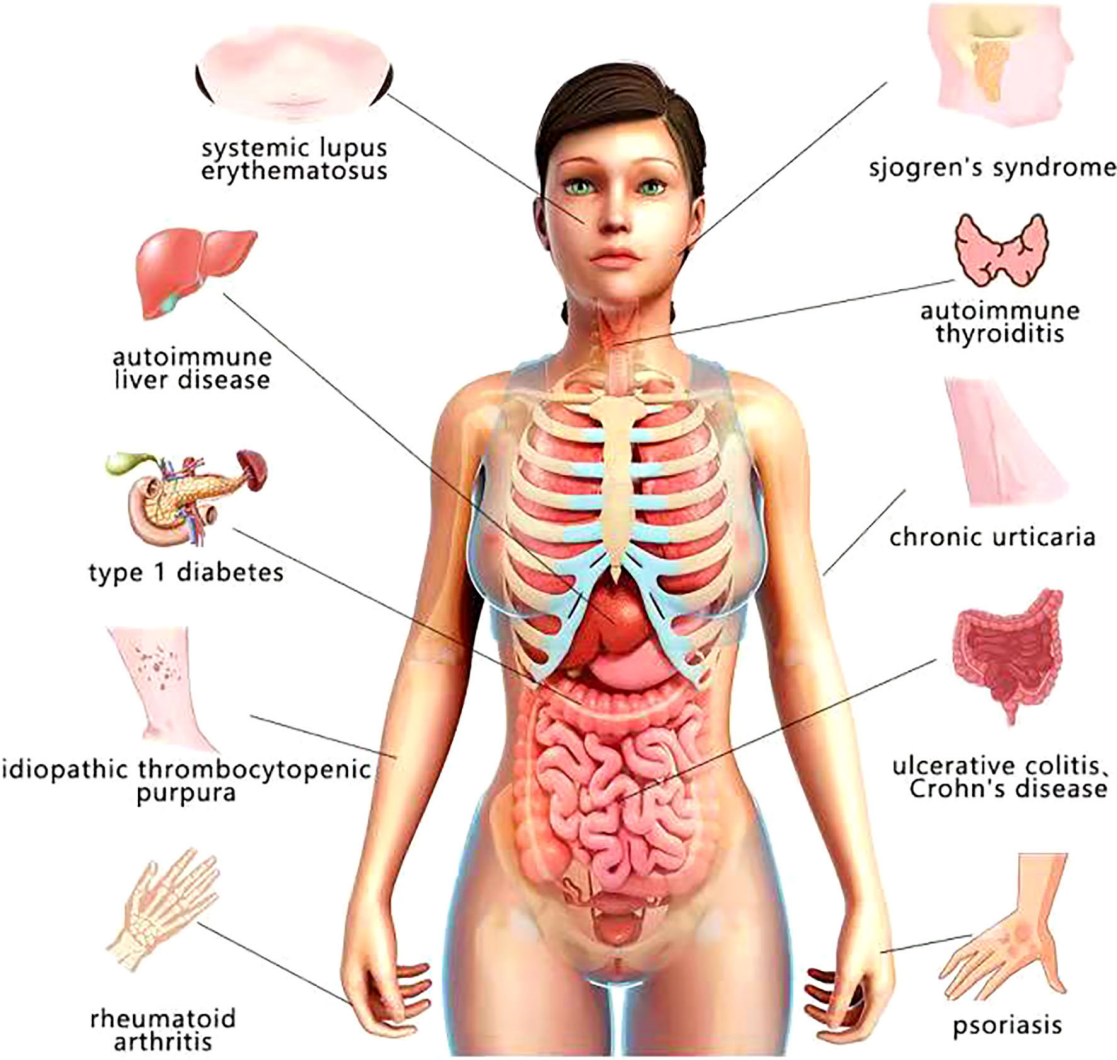
Autoimmune diseases which are likely to have relationship with *H. pylori*.

Autoimmune disease (AD) is a chronic disease usually resulted from the loss of immune tolerance to autoantigens and it can affect a single organ or multiple organs and systems. And genome-wide association researched have shown that the pathological process of AD is affected by the interaction of multiple factors (25). At present, the treatment of some refractory AD still has a large space for development, so it is necessary to explore the pathogenesis and mechanism of AD. Various microorganisms have been discovered to be involved in autoimmunity (26). Infectious agents induce autoimmunity in two ways. First, it provides a homologous antigen-specific signal through molecular simulation or mobilization of endogenous antigens. At the same time, it causes inflammation, producing antigen-specific signals that enhance the immune response through a so-called adjuvant effect (27). The infection of *H. pylori* is among the triggers for many autoimmune diseases (17) (**Figure 2**).

### 3.1 Inflammatory Bowel Disease

Inflammatory bowel disease (IBD) contains a complex group of diseases, among which there are three main phenotypes–Crohn’s disease(CD), ulcerative colitis(UC), and unclassified IBD(IBDU) (28). Over time, sustained chronic intestinal inflammation can lead to tissue injury, including fistulizing and stricturing disease and life-threatening acute onset of severe UC (28). IBD is a major health care problem, with increasing prevalence all around the world (29).

The epidemiological literature generally supports a negative correlation between *H. pylori* and IBD (30, 31). In general, gastrointestinal pathogens are thought to be environmental triggers for new IBD and outbreaks of existing ones. But some bacterial pathogens like *H. pylori* and parasites like trichinella spiralis are negatively correlated with IBD (32, 33). Zhao Hui et al. (34) got the conclusion that *H. pylori* is negatively correlated with IBD, especially CD. Rokkas et al. (35) showed that among the patients, 26.5% of them are infected with *H. pylori*, in contrast with 44.7% of the controls through meta-analysis. Analysis of Asian people, Xiao Wei et al. (36) got the same conclusion. A meta-analysis of 5497 patients, involving 2055 patients with CD and 3442 controls, showed 450 (30.6%) *H. pylori* positive patients with CD and 1476 (42.9%) *H. pylori* positive controls, and the analysis found that *H. pylori* infection was negatively related to the incidence of CD (37). After generally reviewing the meta-analyses, Piovani et al. figured out that *H. pylori* may lower the risk of IBD (38). Yufen et al. (39) integrated epidemiological data for the purpose of detecting the interrelationship between *H. pylori* and IBD and found that IBD, UC, and CD are negatively related to *H. pylori* prevalence. *H. pylori* infection has no connection with the medication and classification of IBD. In addition, the elimination of *H. pylori* may result in the relapse of IBD (39). But the research of Shinichiro et al. showed that eliminating *H. pylori* wouldn’t affect the short-term disease activity of IBD (40). Another case-control study involving 127 IBD patients, among whom, 90 were CD patients, 37 were UC patients, and 254 were controls, got similar conclusion to Shinichiro’s (41). The subtype (more protection against CD than UC) and area (more protection in East Asia than Mediterranean areas) might lead to a variation on the protective effect of *H. pylori* against IBD (42). Zhao Min et al. included 255 clinical studies for meta-analysis and found that *H. pylori* infection can lower the risk of IBD in different geographical populations (43).

Amnon et al. found that this may be in connection with reduced function of gastric acid barrier caused by the infection of *H. pylori*, which protectively affects IBD’s progression (44). An animal experiment showed that *H. pylori*—specific protection against IBD relies on the NOD-like receptor protein 3(NLRP3) inflammasome and IL-18 (45). The protective effect of *H. pylori* infection on IBD may be due to the down-regulation of pro-inflammatory immune response by microorganisms (46). A growing body of data supports this kind of protection, which may be mediated by expression of stron-specific components, particularly CagA (47). But other scholars have found evidence for a significant association between the decreased incidence of IBD(particularly CD) and CagA seropositive *H. pylori* exposure, but not for CagA seronegative *H. pylori* exposure (47). Tepler et al. conducted a meta-analysis of 1,748 people including 960 IBD (688 CD, 272 UC), suggesting that CagA seropositivity was bound up with a lower risk of IBD. CagA seropositivity was related to a 69% reduction in the incidence of IBD and a 75% reduction in the incidence of CD compared with CagA seropositivity (47).

In conclusion, most studies support a negative association between *H. pylori* and IBD, but some scholars suggest that only CagA seropositive *H. pylori* exposure may be relevant to IBD. Whether the eradication of *H. pylori* leads to IBD needs further research.

### 3.2 Autoimmune Metabolic Diseases

#### 3.2.1 Autoimmune Thyroid Disease

Autoimmune thyroid disease (AITD) is a group of autoimmune diseases that regathered thyroid as the target organ, including Graves’ disease(GD), Hashimoto’s thyroiditis(HT), postpartum thyroiditis, etc. Its pathological mechanisms include local infiltration of lymphocyte T cells and B cells, activation of apoptotic gene ligand (Fas-L), the release of various cytokines, as well as cell and tissue inflammation and injury. In addition, cytotoxic effects of thyroid peroxidase antibody(TPOAb) and Tg Ab also jointly lead to the damage and apoptosis of thyroid cells. Its clinical manifestations mainly include diffuse goiter, thyroid pain, thyroid nodules and some pat; some may show symptoms of thyrotoxicosis or hypothyroidism. AITD is related to multiple factors, among which gene and environment are the main ones in the aspect of etiology (48). According to many studies, *H. pylori* is not only in connection with gastrointestinal diseases but also closely related to endocrine system diseases (49).

Whether *H. pylori* is one of the factors causing AITDs is a controversy. A study showed that the TPOAb titers and TgAb titers of 5 AITD patients who were *H. pylori* positive decreased to varying degrees after *H. pylori* elimination, while the TPOAb titers and TgAb titers of AITD patients without *H. pylori* eradication as the control group remained unchanged. It was found that infection of *H. pylori* is likely to be an essential trigger for AITDs (50). Another study from Iran revealed a certain link between the infection of *H. pylori* and HT (51). A review pointed out that the infection of *H. pylori* is likely to trigger AITDs, according to researches of relevant literature (52). A large cross-sectional study suggests that the infection of *H. pylori* is likely to affect the progression of autoimmune thyroid disease. Meanwhile, patients with AITD are also susceptible to *H. pylori* (53). As a support, a meta-analysis including 15 studies and 3,046 patients has also proven that AITD patients have a higher incidence of *H. pylori* than people without AITD, and the elimination of it helps lower the associated autoantibodies (54).A study from Iran showed that only patients infected with the CagA positive strain showed an association with AITDs (55). And another study found similar results (56). Contrary to the above results, a randomized controlled trial showed *H. pylori* has nothing to do with HT (57). Another study revealed that in AITD patients and controls, the infection of *H. pylori* had an effect on the lack of the TPOAb titers and TgAb titer. It shows that a connection between the infection of *H. pylori* and HT is less obvious, compared with the results observed previously (58). Although there are literatures do not support the association between *H. pylori* and AITD, it is reasonable to believe that *H. pylori* infection is bound up with the incidence of AITD based on the results of the meta-analysis mentioned above.

#### 3.2.2 Type 1 Diabetes Mellitus

Type 1 diabetes mellitus (T1DM) is mainly caused by certain factors (infection, chemical poison, diet, etc.), which act on individuals with genetic susceptibility, activate a series of autoimmune diseases mediated by T lymphocytes, and result in the destruction and failure of selective islet β cells and progressive aggravation of insulin secretion deficiency in the body. Clear infection factors are mainly viral infection, including rubella virus, Coxsackie virus, cytomegalovirus, and so on (59). Whether *H. pylori* is relevant to diabetes is still under debate. The infection of *H. pylori* may play an important role in metabolic abnormality (60). Simon et al. (61) first explored the relationship between *H. pylori* and diabetes in 1989. Zekry et al. (62)found that in contrast with the control group, there are much more *H. pylori* IgG in patients with T1DM. According to the studies, T1DM may be related to the increased rate of *H. pylori* infection. Bazmamoun et al. (63) got the result that 48(60%) patients with T1DM were *H. pylori* positive, while 32(40%)patients without diabetes(P=0.030) were *H. pylori* positive, and the incidence of *H. pylori* is positively correlated with the length of the duration of diabetes (P<0.001). Therefore, screening *H. pylori* cases with these follow-ups is helpful. Candelli et al. discovered that younger patients with diabetes are more likely to get reinfected with H. Pylori compared with controls (64). Inflammation resulted from *H. pylori* infection might cause defective insulin secretion. Rahman et al. (65) accounted for a positive connection between the infection of *H. pylori* and impaired insulin secretion. According to a meta-analysis, there is an elevation of glycated hemoglobin A (HbA1c) levels in *H. pylori*-infected patients, in contrast with people not infected (66).

Esmaeili et al. (67), however, drew the opposite conclusion that *H. pylori* infection has no obvious connection with diabetes mellitus in children aged 5-15 years, and there is obvious difference in blood glucose control between T1DM patients who are *H. pylori* positive and those who are *H. pylori* negative. Results of a meta-analysis conducted by Dai et al. suggested that *H. pylori* infection was positively correlated with HbA1c levels in T1DM adolescents and children (68). As shown in a meta-analysis by Li et al. (69), the incidence of *H. pylori* infection is much higher in diabetic patients. This difference is associated with Type 2 diabetes mellitus (T2DM) but not with T1DM. He et al. (70) also put forward a strong association between *H. pylori* and T2DM. Wan et al. found a link between the infection of *H. pylori* and the incidence of diabetes among Chinese adults (71).

At present, there is still no unified conclusion on whether an obvious connection exists between *H. pylori* infection and diabetes. There is more evidence of a link with T2DM. A new study of this has emphasized the cause-and-effect relationships between bacteria and insulin resistance or autoimmunity, and discussed whether the glycemic result of DM patients would deteriorate due to *H. pylori* infection. Studies on this topic still need to be finalized to evaluate and explain the potential role *H. pylori* plays in the cause and course of DM (72).

### 3.3 Autoimmune Liver Diseases

Autoimmune liver diseases(ALD) are clinically common chronic inflammatory hepatobiliary disorders (73). Some researchers believe that chronic liver disease and *H. pylori* are related (74, 75). The residing of *H. pylori* at the top of the gastrointestinal epithelium can directly cause severe damage to the mucosal barrier (76). Invasion of the intestinal mucosa by *H. pylori* may increase intestinal permeability and promote bacterial endotoxin to pass through the portal vein and reach the liver (77). In an immunohistochemical research on *H. pylori*, positive antigen fragments was found existing in the liver of *H. pylori*-positive cases (78). As shown in animal studies, oral *H. pylori* can arrive in the hepatobiliary system and result in inflammation to be an independent cause (79). The infection of *H. pylori* is related to hepatic dysfunction. In addition, *H. pylori* infection can stimulate liver cells and accumulate collagen, which may lead to liver fibrosis (80). It’s even been linked to hepatocellular carcinoma (81). Salehi et al. demonstrated that after receiving *H. pylori* eradication regimens, liver enzymes are reduced (82). It has been found that the patients suffering from HCV or HBV have more possibility to get infected with *H. pylori*, compared with patients with autoimmune liver cirrhosis (ALC) or primary biliary cirrhosis (83). The meta-analysis of Feng et al. includes 21 studies, involving 6135 cases, showing that the cirrhosis patients are more possibly to get infected with *H. pylori* compared with controls. According to etiology, the relatively higher pylori-positive rate is resulted from PBC and viral cirrhosis in contrast with ALC (84). Nilsson et al. (85) found that the prevalence of non-gastric Helicobacter species antibodies in serum of patients suffering from autoimmune chronic liver disease is obviously higher in contrast with controls (P<0.001).

#### 3.3.1 Primary Biliary Cirrhosis

Primary biliary cirrhosis (PBC) is a liver disease mediated by an autoimmune response. The pathological manifestations are chronic progressive non-suppurative cholangitis or granulomatous cholangitis with the destruction of small bile duct rupture. The clinical manifestations are relatively hidden but they are accompanied by a continuous rise in alkaline phosphatase(ALP), and anti-mitochondrial antibodies (AMA) can be detected in 80-95% of PBC patients (86, 87). The antigenic molecules recognized by AMA are mainly pyruvate dehydrogenase complexes. In addition, abnormal expression of human leukocyte antigen DR(HLA-DR) and DQ antigen molecules in biliary epithelial cells leads to T lymphocyte-mediated cytotoxicity, which continues to damage the canaliculi.

Goo et al. (88) reported a case of PBC, in which a mouse was infected with *H. pylori* in 2008; however, this case had significant histological similarities to human PBC. Therefore, they believed that the increase in vacuolar toxin resulted from *H. pylori* infection may have a correlation with the occurrence of PBC through molecular simulation. Abenavoli et al. (89) reported a woman diagnosed with PBC, *H. pylori* infection, and celiac disease. After sticking to a diet without gluten strictly, being treated in association with the management of ursodeoxycholic acid(UDCA) and the elimination of *H. pylori*, the woman got a much better status in clinic. Their experience supported the pathogenic role of increased intestinal permeability in inducing PBC in celiac disease and *H. pylori* infection. *H. pylori*-positive rate is obviously higher in patients with PBC and PSC(20 of 24), compared with those without cholestatic diseases and healthy controls (1 of 23) (90). Specific interactions of infections possibly increases PBC risk. There exists a much higher prevalence of four anti-infectious agents Abs in patients with PBC than in controls, namely anti-T.gondii Abs(ATxA)(71% vs. 40%, p<0.0001), *H. pylori* (54% vs. 31%, p<0.01), EBV early antigen (EBV-EA)(44% vs. 12%, p<0.0001), and cytomegalovirus(CMV) (90% vs. 75%, p<0.05) Abs, respectively. The coexistence of the four anti-infective Abs is a very common phenomenon in patients with PBC, and this infection burden is less seen in normal people (20% vs. 3%, respectively, P <0.0001) (91). The mitochondrial autoepitopic area of pyruvate dehydrogenase complex E2 (PDC-E2) resembles urease β of *H. pylori*, which means that *H. pylori* infection has something to do with the incidence of PBC (92). Since the existence of the DNA of pylori was first discovered in liver tissue, antibodies to the microbe in the bile and the serum of patients who suffered from PBC, the researchers have listed it in the pathogenesis of PBC (93).

#### 3.3.2 Primary Sclerosing Cholangitis

Primary sclerosing cholangitis(PSC) is an idiopathic progressive chronic intrahepatic cholestasis, which is thought to be caused by fibrostenosis of the extrahepatic bile duct. It is a relatively rare disease whose pathogenesis and treatment remain uncertain. It has a poor prognosis and leads to biliary cirrhosis, which causes liver failure (94). There is usually a correlation between PSC and IBD, especially ulcerative colitis (UC). Long-term monitoring of patients with PSC highlights an increased incidence of biliary, gallbladder and colon cancers, which may be associated with chronic inflammation and bile acid exposure (95). Since PSC’s inflammatory process and gastric metaplasia are similar to chronic gastritis induced by *H. pylori*, we investigated its association with *H. pylori* (96). Krasinski et al. (96) studied 25 patients who were at the end stage of PSC and 31 controls and found that 7 of 25 PSC patients (28%) and 3 of 31 controls (9.7%) were *H. pylori* positive (P=0.087). The PSC patients are more likely to get infected with *H. pylori* in the microdissected hilar biliary epithelium compared with controls, which supported the assumption when bile reflux happens from the duodenum into the biliary tract. *H. pylori* may be carried into the proximal biliary system, thus PSC may develop and/or progress in certain patients. And those patients with PSC are likely to develop ulcerative colitis (UC), from which a suggestion has been made that the *H. pylori* translocation is likely to be promoted as a result of the increase of intestinal permeability in UC patients to the hepatobiliary system, thus triggering autoimmune mechanisms (97).

#### 3.3.3 Autoimmune Hepatitis

Autoimmune hepatitis (AIH) is a rare idiopathic syndrome characterized by the liver-cell destruction from immune mediation, which is mainly related to autoantibodies. The cause of the disease is not fully comprehended (98). The disability in tolerating hepatic antigens is considered to be caused by environmental pathogens which include xenobiotics and pathogens in genetically susceptible individuals (99). So *H. pylori* may be one of the pathogens. There is growing evidence that the gut microbe biota, which contains more genes than the human genome, has come to be a pivotal environmental trigger for liver disease along the gut-liver axis (100–102). The importance of the liver-microbiome axis is more and more recognized to be a significant regulator of autoimmunity (103). *H. pylori* causes inflammation and changes the stomach and gut microbiota (104). Therefore, *H. pylori* may also promote pathogen translocation and lead to liver autoimmunity by affecting intestinal flora. Although the DNA of *H. pylori* has been found in hepatic tissues of a small number of AIH patients, no significant difference has been found in comparison of these patients and controls (105). Durazzo et al. (106) found that patients and controls have similar rates of *H. pylori* infection. At present, there are few literatures about the correlation between AIH and *H. pylori*, and further studies are needed.

### 3.4 Rheumatic Diseases

#### 3.4.1 Rheumatoid Arthritis

Rheumatoid arthritis(RA), which includes synovitis, cartilage damage, and symmetry joint damage as main clinical manifestations, often occurs in small joints such as hands and feet and even leads to joint deformity and loss of function. It can even involve other systems outside the joint, including pericarditis, pulmonary fibrosis, peripheral neuropathy, and other diseases. The global prevalence of RA is 0.24% (107), causing substantial morbidity and increased mortality with annual costs of billions of dollars (108). Several factors have been listed as the clinical predictors of the incidence of rheumatoid complication, including high pro-inflammatory markers levels, smoking habit, high titer of rheumatoid, worse function, male gender, and severe joint disease (109). According to current data, the early stages of RA are characterized by an early response to a limited number of autoantigens, as well as limited systemic inflammation, followed over time by innate and adaptive responses and evolutionary damage to tissues, until a certain threshold is exceeded and clinically evident RA appears (19). So is the accumulation of immune response caused by *H. pylori* related to RA? Can it be predicted by one of the factors?

The possibility of the correlation between *H. pylori* and RA is still controversial. The vitro researches have identified urease, which stimulates B-1 cells to produce various autoantibodies such as IgM rheumatoid factor (19). Ebrahimi et al. (110)found that the levels of rheumatoid factor(RF), erythrocyte sedimentation rate(ESR), C-reactive protein(CRP), and anti-cyclic peptide containing citrulline (anti-CCP) antibody within RA patients with *H. pylori* are obviously higher than those in RA patients without *H. pylori* infection. Das-28 and VAS scores are obviously higher in RA patients with CagA+ than in CagA- patients. Zeitlin et al. (111) also showed similar results that laboratory indicators such as ESR and C-reactive protein in *H. pylori*-positive patients are higher than *H. pylori*-negative patients, and clinical manifestations such as joint pain and dysfunction are more obvious in *H. pylori* positive RA patients. The infection of *H. pylori* can trigger or aggravate AID (112), and the eradication of *H. pylori* can also improve the clinical efficacy of rheumatoid arthritis to a certain extent. Some scholars conducted a study on 140 sufferers with active RA and found that the symptoms of patients in the radical treatment group were improved, and the ESR, CRP, IL-8, and IL-18 were at lower levels than those in the control group (113).

Nevertheless, other studies have shown the opposite. Bartels et al. (114) have found the infection of *H. pylori* in Denmark has no relation with RA. In 187 samples from patients with RA, 80.4% of them were infected by *H. pylori*; however, the incidence of *H. pylori* infection of healthy controls was 80.7% (115). Compared with healthy Japanese people, the level of *H. pylori* antibody in RA patients was lower (116), which may probably reflect interpopulation variance. In Youssefi M.’s systematic review and meta-analysis suggested no apparent correlativity between the infection of *H. pylori* and RA (117). *H. pylori* infection had no remarkable influence on the onset of RA (117). So, the relevance between *H. pylori* and RA is population-based, and the results may also be affected by sample size and region.

Although the correlation between *H. pylori* and RA is under debate, RA patients may continue to take NSAIDs. If combined with *H. pylori* infection, gastric mucosa can be synergically damaged, and gastric ulcer bleeding can be induced. Therefore, *H. pylori* should be monitored regularly in RA patients.

#### 3.4.2 Immune Thrombocytopenic Purpura

Immune thrombocytopenic purpura(ITP) is another kind of autoimmune-mediated disease featured by autoreactive antibodies produced by immune dysregulation of T and B cells, resulting in defective platelet clearance and formation. Now, it is still hard to fully figure out the causes of the disease. In the existing studies, the pathogenesis of ITP involves many aspects, including common humoral immunity, cellular immunity, etc. In recent years, more and more evidences manifest that the infection of *H. pylori* is relevant to nosogenesis of ITP. ITP triggered by the infection of *H. pylori* has been proved by plenty of mechanisms put forward, including molecular mimicry resulted from the generation of autoantibodies against CagA and the cross-reactivity of the antibodies with platelet surface antigens, phagocytic perturbation by enhanced phagocytic activity of monocytes, platelets aggregation on account of the existence of anti-*H. pylori* IgG and von Willebrand factor (vWf), and ultimately the parasitifer immune response against CagA and VacA, bringing about ITP (118).

Researches have shown that an obvious improvement of platelet counts is observed in the groups with *H. pylori* infection and after eradication therapy, in contrast with the group without *H. pylori* and those infected with *H. pylori* but without elimination treatment (119). Another research evaluated the role *H. pylori* elimination monotherapy played on the recovery of platelet count in ITP patients, which showed that ITP patients who successfully eradicated *H. pylori* have increased platelet counts, but persistent *H. pylori* infection responds poorly to platelet recovery due to failed eradication attempts (120). In addition, in children and pregnant women groups, it also get effective verification. As shown in the first randomized research in Latin America, it is of significant importance to examine whether the children and adolescents with ITP are infected with *H. pylori* when dealing with the disease; the PLT count is obviously higher in patients who get treated (121). Data from 706 children in 18 previous reports showed a *H. pylori* infection rate of 23% and a platelet response rate of 43.8%, and the effect of *H. pylori* eradication in children is the same as that in adults (122). Some scholars treated pregnant women for *H. pylori*-positive ITP after purging *H. pylori*. In three of the four cases, platelet counts rose to levels in excess of 10x109/L two weeks after eradication, and these levels were maintained until delivery (123). To sum up, it is of significant effect to increase the platelet count of patients with ITP by eradicating *H. pylori*; the *H. pylori* eradication may inhibit the production of anti-platelet autoantibodies, and many patients’ diseases can be alleviated or even cured (124, 125).

Because *H. pylori* is closely related to ITP, every patient with unexplained thrombocytopenia should go through *H. pylori* test. If the result shows *H. pylori* positive, *H. pylori* eradication therapy must be tried in every ITP case, or the conventional ITP therapies won’t have an effective treating result on the patients (126).

#### 3.4.3 Systemic Lupus Erythematosus

Systemic lupus erythematosus (SLE) is a systemic AID featured by inflammation and abnormal generation of autoantibodies, which are anti-double-stranded DNA antibodies(anti-dsDNA) and antinuclear antibodies(ANA). It may cause especially serious organ damage and complications, including but not limited to pneumonia, nephritis, encephalitis, myelitis, mesenteric vasculitis, and thrombocytopenia, and in severe cases, irreversible damage or even death (127). In China, the prevalence of SLE is about 30/100,000 to 70/100,000 (128). The exposure of genetically susceptible populations to some exterior factors, such as viruses, bacteria and protozoa, may act as catalysts for SLE initiation. Among all the infectious pathogens raised to induce autoimmunity, *H. pylori* is among the subjects which are researched most (129). A cohort research got the conclusion that the infection of *H. pylori* is correlated to a 1.63-fold increased risk of SLE, especially in women <30 years of age (130). Early elimination of *H. pylori* significantly reduces the possibility of SLE during the three-year follow-up period (131). The previous researches have indicated that in SLE patients, the incidence of *H. pylori* infection is as high as 39%, but the severity of SLE seems unrelated to the infection of *H. pylori*, and immunosuppressive therapy might be ineffectual in preventing *H. pylori* infection in patients with SLE (132). Defects in the Fcγ receptor IIb(FcγRIIb) have been identified as genetic factors that increase susceptibility to lupus; the previous experiment on mice showed that *H. pylori* infection increases anti-dsDNA and promotes anti-dsDNA lupus seriousness in mice with the symptom of FcγRIIb-deficient lupus (133). Thus, an increased systemic inflammatory response caused by localized *H. pylori*-induced gastritis may accelerate lupus progressions in some lupus patients. The connection between *H. pylori* infection and autoimmune disease is of multiple facets and directions, including pathogenicity and/or protective associations (134).

But there are studies that show the opposite. Basing on the concomitant growing occurrence in autoimmune diseases and decreasing occurrence in infections, researchers developed the hygiene hypothesis, which suggested that *H. pylori* infection might have a protective influence on autoimmune diseases (135). The possible protective mechanisms of infections include: First, antigenic competition results in reduced responses to their own antigens; Second, inhibition of parasitifer immune response to self or non-self molecules by stimulating Treg cell subsets; Third, Toll-like receptors(TLR) signals regulate immunosuppression of Treg cells in direct or indirect ways (136). The correlation between serologically negative *H. pylori* and SLE development in African American women suggested that infection of *H. pylori* may protectively affect the SLE pathological process or *H. pylori* serologically positive immunomodulatory events are negatively associated with SLE risk (137). The existing meta-analyses and systematic reviews show that no apparent association exists between the infection of *H. pylori* and SLE susceptibility (117). In summary, in short, there is no definite conclusion. The correlation between these two objects needs further study.

#### 3.4.4 Sjogren’s Syndrome

Sjogren’s syndrome(SS) is featured with lymphocytic infiltration happened to exocrine glands and other apparatuses, which have connection with the generation of all kinds of qutoantibodies in the blood. Mostly, this kind of disorder progresses secretly for maybe as short as several months or as long as several years. Its typical symptoms include dryness of eyes and mouth, fatigue, and dilatation of the major salivary glands. It is found that, in some cases, when the exocrine glands suffer the lesion and malfunction of exocrine glands, which happens gradually and progressively, the whole-body dryness may be caused, and it’s called the sicca syndrome (from the Latin siccus meaning dry or thirsty) (138–141). SS mostly affects women, and symptoms usually occur during the patient’s fifth or sixth decade of life (142). Among all the cases, 25% of them may involve internal organs, and the major complication of SS is the progression of non-Hodgkin’s B-cell lymphomas.

Some researchers has found that SS, with the typical symptoms including lymphocytic infiltration and damage of exocrine glands, has a link with *H. pylori*. Qianqian et al. (143) did a meta-analysis involving 1958 patients in 9 studies, in which 619 patients were with SS. Among all the patients, 53.83% of them were infected with *H. pylori*, and *H. pylori* infection rate among the SS sufferers were much higher, in contrast with the controls. El Miedany et al. (144) found that compared with healthy people and other connective tissue disease patients, the SS patients have a higher infection rate of *H. pylori*. It was also found that the prevalence of *H. pylori* antibodies in the SS patients is higher than the controls, after evaluating the existence of *H. pylori* antibodies in Italians with anti-Ro positivity antibodies (145). Moreover, a research (146) which involved 118 people, who were separated in four groups (primary SS, secondary SS, other AID, and healthy controls), showed that the frequency of serum antibodies against *H. pylori* increased in primary SS patients in contrast with the other three groups.

This phenomenon may be due to an autoimmune response which is induced by bacteria, and *H. pylori* is a notorious cause for autoimmune responses. But, according to some researches, no obvious disparity in the incidence of *H. pylori* infection exists between SS and the control group. As shown in a research involving 164 patients with primary SS in Sweden, the result showed that there was no distinction in seropositive rate between the *H. pylori* positive patients and controls (17). Another clinical study showed that while there was no imparity in the infection rates of *H. pylori* between patients with SS and those without SS, and 90% of the patients were infected with CagA positive strain, in comparison with 37% of the infected controls (147). Hence, no agreement has been reached on whether *H. pylori* infection is a trigger for SS or not. Perhaps CagA positive strain is closely related to SS.

### 3.5 Autoimmune Dermatosis

#### 3.5.1 Chronic Urticaria

Chronic urticaria (CU) is one of the common illness driven by mast cells, with the clinical manifestations of the sudden occurrence of urticaria and/or angioedema, erythema, severe itching, which is self-limited and easy to repeated attacks. Chronic urticaria is segmented into CU, chronic spontaneous urticaria(CSU), and chronic inducible urticaria(CINDU) (148). Chronic urticaria is characterized by both an autoimmune and allergic disease profile. The current pathogenesis is not completely understood (149). There are researches which have probed the correlation between the infection of *H. pylori* and CU. A clinical research including 55 CU patients and 55 controls indicated *H. pylori* infection has an influence on CU, and CU patients are of an obviously higher rate of *H. pylori* infection than control subjects (150). Another meta-analysis in which 965 CU patients and 1235 controls were involved in 16 clinical trials indicated that, though weakly, the infection of *H. pylori* is of an obvious effect on the increased risk of CU (151). A meta-analysis of 1320 patients enrolled by Magen et al. showed that the infection of *H. pylori* was related to the development of CU (152). Meanwhile, relevant researches have found that the elimination of *H. pylori* can do help to evaluate the prognosis of CU and may reduce the recurrence rate of chronic urticaria (153, 154). On the contrary, a study including 74 CU patients who were *H. pylori* positive and 74 controls demonstrated that neither the infection of *H. pylori* nor its eradication therapy will affect the clinical course of CU. *H. pylori* infection is possibly related to the onset and persistence of chronic spontaneous urticaria(CSU), and the efficacy of *H. pylori* elimination in ameliorating CSU symptoms is remarkable. Interestingly, CSU patients who accepted the treatment of antibiotic to eradicate this kind of bacillus had visibly higher CSU response rates, regardless of whether *H. pylori* was eradicated (155). That is to say, whether *H. pylori* is related to CU is not sure (156). We recommend that promoted studies pay more concentration to probing the mechanisms related to the correlation between *H. pylori* and CU.

#### 3.5.2 Psoriasis

Psoriasis is a kind of chronic immune-associated recurrent inflammatory dermatosis whose typical clinical manifestations is scales erythema. It is currently believed that immune cell activation, keratinesis, and abnormal cell differentiation are key to the pathogeny of psoriasis (157). The association between the infection of *H. pylori* and psoriasis is relatively contradictory. Some studies show that psoriasis has no obvious correlation with *H. pylori* antibody level, and slight to serious psoriasis patients haven’t demonstrated a higher incidence of *H. pylori* infection (158, 159). Therefore, further researches should be made with the purpose of figuring out whether *Helicobacter pylori* infection is among the factors which may lead to or deteriorate psoriasis. There have also been relevant studies showing that the *H. pylori* infection may affect the seriousness of psoriasis, and eliminating this infection can improve the effectiveness of psoriasis treatment (160–162).

## 4 Conclusion and Expectation

The current paradigm for the development of autoimmune diseases is understood to be that they are triggered by exposure of genetically susceptible individuals to environmental factors (163). According to the studies above, we can confirm that the infection of *H. pylori* is a significant one among many environmental factors which lead to many types of autoimmune diseases. Therefore, we believe that the occurrence and development of autoimmune diseases may be promoted in populations with genetic susceptibility to autoimmune diseases under the action of helicobacter pylori infection as an exposure factor. In conclusion, it is negatively correlated with IBD, possibly positively correlated with PBC, PSC, and ITP. There are many literatures supporting that the infection of *H. pylori* may be relevant to AITD and CU, while there are few literatures about AIH and Psoriasis. The correlation between the infection of *H. pylori* and the occurrence of RA and SLE need to be further studied. The correlativity between the infection of *H. pylori* and T1DM is uncertain, but more and more evidences show that the infection of *H. pylori* is strongly related to T2DM. **Table 1** provides a quick summary. Several literatures indicate that CagA+ *H. pylori* strain is associated with the incidence of various autoimmune diseases (164), and the existence of CagA antigen is possibly a main element affecting the level of autoantibodies (165).In the future, it is necessary to find and prove the exact *H. pylori* antigen has one homologous domain with a specific human antigen, figure out a cause-and-effect relationship between the pathogenesis of different autoimmune diseases and the infection of *H. pylori*, put across the clinical significance of *H. pylori* on the risk and progression course of different autoimmune problems, and explore the possible common signaling pathways that exist in different autoimmune diseases caused by the infection of *H. pylori*, the possible therapeutic or preventive benefits as well.

**Table 1:** Evidences for or against *H. pylori* associated with autoimmune diseases.

Evidences for or against *H. pylori* associated with autoimmune diseases			
Disease	Ref.	Study Sample	Conclusion
IBD	Wang WL et al. 2019 (37)	2055 CD patients and 3442 controls for meta-analysis were included.	*H. pylori* infection was negatively associated with the incidence of CD
IBD	Yufen et al. 2021 (39)	59 studies on IBD prevalence, 127 studies on *H. pylori* prevalence, and 23 studies for meta-analysis were included.	IBD, UC and CD were negatively correlated to *H. pylori* prevalence (all P<0.001). *H. pylori* had a protective effect against IBD. Furthermore, eradication of *H. pylori* can lead to recurrence of IBD.
IBD	ZhaoHui et al. 2021 (34)	213 CD patients and 47 UC patients, and 520 controls	*H. pylori* infection was negatively correlated with IBD, especially CD.
IBD	Rokkas et al. 2015 (35)	4763 controls and 4400 IBD patients	26.5% of IBD patients were positive for *H. pylori* infection, compared with 44.7% of the control group.
IBD	Piovani et al. 2019 (38)	183 estimates in 53 meta-analyses of 71 environmental factors related to hygiene and lifestyles, surgeries, diet, microorganisms,etc.	*H. pylori* reduced the risk of IBD, Non-helicobacter pylori-like enterohepatic Helicobacter species increase the risk.
IBD	Zhao M et al. 2021 (43)	255 clinical studies for meta-analysis	*H. pylori* infection can reduce the risk of IBD in different geographical populations
IBD	Amnon et al. 2020 (44)	302,061 patients, of whom 13,943 harbored IBD	Reduced gastric acid barrier function may have a protective effect on the development of IBD.
IBD	Wang WL et al. 2019 (37)	2055 cases of CD patients and 3442 cases of controls	*H. pylori* infection was negatively associated with the incidence of CD
IBD	Rosa et al. 2018 (41)	127 IBD patients (CD_N_=90; UC_N_=37) and 254 controls	*H. pylori* eradication therapy was not associated with the onset of IBD.
IBD	Tepler A et al. 2019 (47)	960 IBD patients(CDN=688,UCN=272)and 788 controls	CagA seropositivity was associated with a lower risk of IBD
AITD	Bertalot G, et al2004 (50)	10 patients with *H. pylori* infected [all females, mean age 46 years (SD 19 years)]	the thyroid gland is a target of a cross-reaction in the human immune response to *H. pylori* infection.
HT	Francesco F, et al2004 (58)	16 patients (two men and 14 women, mean age 43.6 ± 11 years) with HT and *H. pylori* infected 20 blood donors (two men and 18 women, mean age 44.2 ± 12 years) without HT was also evaluated.	an association between *H. pylori* infection and HT is unlikely.
AITD	Choi YM, et al2017 (53)	5502 subjects aged 30 to 70 years who had visited a health promotion center	prevalence of TPO-Ab positivity is more frequent in subjects with *H. pylori* infection.
AITD	Hou Y, et al2017 (54)	3046 patients diagnosed with HT or GD	*H. pylori* infection correlated with GD and HT, and the eradication of *H. pylori* infection could reduce thyroid autoantibodies.
HT	Shmuely H, et al2016 (57)	101 females with HT and 111 non-HT control women without a history of autoimmune disease.	H pylori seropositivity was not associated with HT.
AITD	Soveid M, et al2012 (55)	88 patients with ATD and compared with results of 112 healthy individuals.	in a population with high rate and early age of onset of HP infection, only infection with Cag A positive strains is associated with ATD, and this may be due to immune cross-reactivity.
HT&GD	Bassi V, et al. 2012 (56)	112 consecutive Caucasian patients (48 females and 4 males with Graves' disease and 54 females and 6 males with Hashimoto's thyroiditis HT)	the marked correlation between *H. pylori* and Cag-A, found in ATDs, could be dependent on the different expression of adhesion molecules in the gastric mucosa.
HT	Aghili R, et al. 2013 (51)	43 patients affected by Hashimoto's thyroiditis, and 40 healthy individuals without history of autoimmune disease as the control group.	there is an association between HP and Hashimoto's thyroiditis.
T1DM	Zekry et al. 2013 (62)	60 children and adolescents with T1DM and 60 controls	T1DM can be associated with an increased prevalence of Hp infection.
T1DM	Bazmamoun et al. 2016 (63)	80 children with T1DM and 80 controls	There was a meaningful correlation between the frequency of *H. pylori* and the longer the duration of diabetes (P<0.001).
T1DM	Chen et al. 2019 (66)	35 studies with 4,401 participants with diabetes	Glycated hemoglobin A levels were elevated in patients with *H. pylori* infection.
T1DM	Esmaeili et al. 2020 (67)	63 children with T1DM and 105 control children	T1DM and its glycemic control levels did not appear to be associated with *H. pylori* infection.
T1DM	Li et al. 2017 (69)	57,397 individuals with *H. pylori* infection in DM individuals and non-DM individuals (individuals without DM, impaired glucose tolerance, or impaired fasting glucose)	The incidence of *H. pylori* infection was significantly higher in diabetic patients, however that was associated with T2DM but not with T1DM.
T1DM&T2DM	Dai YN et al. 2015 (68)	513 patients with diabetes mellitus (DM) and 325 T2DM participants	*H. pylori* infection was positively correlated with HbA1c levels in T1DM children and adolescents
LC	Pogorzelska et al. 2017 (83)	147 patients with liver cirrhosis: 42with HCV, 31 with HBV, 56 ALC, and 18 PBC	*H. pylori* infection is more frequent among patients with HBV and HCV.
LC	Feng et al. 2014 (84)	6135 patients divided into cirrhosis group and the control group	There are significant difference in *H. pylori* infection between patients with cirrhosis and controls.
AILD	Nilsson et al. 2003 (85)	36 PSC,21 PBC,19 AIH and 80 controls	The prevalence of non-gastric Helicobacter species antibodies in serum of patients with autoimmune chronic liver disease was significantly higher than that in controls(P<0.001).
PBC	Goo et al. 2008 (88)	a 24-month-old male mouse	The increase in vacuolar toxin caused by *H. pylori* infection may be related to the occurrence of PBC.
PBC	Abenavoli et al. 2010 (89)	a 36-year-old woman	The pathogenic role of increased intestinal permeability in inducing PBC in celiac disease and *H. pylori* infection.
PBC and PSC	Nilsson et al. 2000 (90)	24 patients and 23 controls.	Helicobacter positivity was significantly more common in patients with PBC and PSC.
PSC	Krasinskas et al. 2007 (96)	25 patients with end-stage PSC and 31 controls	7 of 25 PSC patients (28%) and 3 of 31 controls (9.7%) were *H. pylori* positive.
AIH	Durazzo et al. 2002 (106)	31 patients and 62 controls.	Patients and controls had similar rates of *H. pylori* infection.
RA	Ebrahimi et al. 2019 (110)	100 patients with RA	The RA laboratory index activity of *H. pylori* positive patients was higher than that of *H. pylori* negative patients.
RA	Zentilin,P., et al. 2002 (111)	58 adult patients with established rheumatoid arthritis and dyspeptic symptoms	*H. pylori* infection is implicated in the pathogenesis of RA.
RA	Guimei L et al. 2015 (113)	140 patients with active RA	Eradication of *H. pylori* can improve the clinical efficacy of rheumatoid arthritis to a certain extent.
RA	Bartels et al. 2019 (114)	56000 people diagnosed as *H. pylori* positive or negative.	They had similar rates of comorbidity. No link was found between *H. pylori* and RA.
RA	Cross M ,et al. 2010 (107)	187 samples from RA patients	There was no significant difference in the positive rate of *H. pylori* between the control group and the experimental group.
RA	Tanaka E,et al. 2005 (116)	1815 patients with RA	The prevalence of *H. pylori* antibody was low in patients with RA compared with that in healthy Japanese individuals.
ITP	Hwang,et al. 2016 (119)	102 patients with chronic ITP	*H. pylori* eradication therapy was related to increased platelet count.
ITP	Brito,et al.,2015 (121)	85 children with cITP	the PLT count was significantly higher in patients with *H. pylori* eradication.
ITP	Ikuse, et al. 2020 (122)	706 pediatric patients with cITP persistent thrombocytopenia (platelet count <150,000/μL) for longer than 6 months	The effect of *H. pylori* eradication in pediatric cITP patients is the same as that in adults.
ITP	Ono, Y., et al. 2017 (123)	4 pregnant women	In pregnant women with HP-associated ITP, eradication of Hp may be worthwhile before steroid use is considered.
ITP	Sheema, et al. 2017 (124)	85 patients diagnosed with chronic ITP	Anti-*H. pylori* eradication therapy improves blood platelet counts in chronic immune thrombocytopenia.
ITP	Aljarad,et al. 2018 (125)	50 patients with chronic ITP	*H. pylori* eradication significantly increases platelet counts in adult ITP patients.
SLE	Wu, M.C., et al. 2020 (131)	41,651 patients with HP infection and 83,302 matched controls	HP infection was associated with a 1.63-fold increased risk of SLE.
SLE	Mendoza-Pinto,C.,et al. 2020 (132)	118 patients with SLE	The frequency of HP infection was up to 39% in patients, but the severity of SLE did not appear to be associated with HP infection.
SLE	Saowapha et al. 2018 (133)	FcγRIIb-deficient lupus mice	HP infection increased anti-dsDNA and enhanced lupus severity.
SLE	Sawalha, et al. 2004 (137)	466 patients with SLE and 466 controls	HP infections may play protective roles in.
SS	Qianqian, et al.2018 (143)	1958 participants, including 619 patients with SS	The patients with SS had a significantly higher *H. pylori* infection rate than controls.
SS	P A , et al.1999 (146)	118 persons	An increase in the prevalence of serum antibodies against *H. pylori* and HSP60 was in primary SS patients compared with the rest of the groups.
SS	Shigenori, N, et al. 2007 (17)	164 patients with primary SS	There is not a higher sero-positive rate of *H. pylori* in patients compared with controls.
CU	Dennis, et al. 2020 (150)	55 CU cases and 55 controls	There was a strong and significant association between CU and *H. pylori* infection.
CU	Gu, H., et al. 2015 (151)	965 CU cases and 1235 controls	*H. pylori* infection is significantly, though weakly, associated with an increased risk of chronic urticaria.
CU	Rasooly, et al. 2015 (153)	204 patients with CU	Eradication of infection can be followed by remission of urticaria, reduced morbidity from gastric ulcers, and cancer.
CU	Magen, et al. 2007 (154)	78 patients with CU	Eradication of *H. pylori* infection by triple therapy significantly and equally reduces UAS in CU patients with positive and negative ASST.
CU	Hellmig, et al. 2008 (156)	74 CU patients with positive *H. pylori* breath test and 74 *H. pylori*-negative controls.	There is no evidence that eradication of *H. pylori* improves the outcome in patients with chronic urticaria.
CU	Cui YL et al. 2021 (152)	1,320 patients with CU	*H. pylori* infection was associated with the development of chronic urticaria (151)
Psoriasis	Wu, et al2020 (158).	41,539 patients with HP infection and 83,078 matched controls	There is no association between HP infection and risk of psoriasis.
Psoriasis	Azizzadeh, et al. 2014 (159)	61 patients with psoriasis vulgaris and 61 healthy individuals	There was neither a significant relationship between psoriasis and the serum level of IgG anti-H pylori, nor a significant relationship between psoriasis severity and the serum level of IgG anti-H pylori.
Psoriasis	Yu, et al. 2019 (160)	11 studies involving a total of 1741 participants	*H. pylori* infection is associated with psoriasis, and psoriasis patients with *H. pylori* infection have higher Psoriasis Area and Severity Index (PASI) scores.
Psoriasis	Campanati, et al. 2015 (161)	210 patients with psoriasis and 150 healthy controls	Patients with mild to severe psoriasis do not show a greater prevalence of *H. pylori* infection; however, *H. pylori* seems able to affect the clinical severity of psoriasis.
Psoriasis	Onsun, et al. 2012 (162)	300 patients with psoriasis and 150 non-psoriatic healthy controls	*H. pylori* infection plays a role in the severity of psoriasis, and that eradicating such infections enhances the effectiveness of psoriasis treatment.

Future research may be carried out from the following perspectives. Researchers can extract strains of *H. pylori* from cohort who suffered the same kind of autoimmune disease in different countries and then conduct whole-gene scans to look for specific genes that may be involved, and thus search for the cause of the autoimmune disease. The specific gene of *H. pylori* will then be knocked out and used to infect a mouse model of the autoimmune disease. Changes in relevant cytokines and antibodies will then be detected. At the same time, comparison with the mouse infection model without gene knockout was conducted to find whether there are specific genes and specific promoters of *H. pylori* to initiate autoimmune diseases.

## Author Contributions

LW, Z-MC, and X-cD designed the review and wrote the article. Z-jL and Y-xZ draw pictures and made critical revisions. W-lL contributed to the critical revision of the manuscript for important intellectual content. All the authors participated the revision of some important parts of the article. LZ, XL, and QW put forward some valuable suggestions in the content of the article.

## Funding

National Key Research and Development Program of China (No. 2018YFC1705700).

## Conflict of Interest

The authors declare that the research was conducted in the absence of any commercial or financial relationships that could be construed as a potential conflict of interest.

## Publisher’s Note

All claims expressed in this article are solely those of the authors and do not necessarily represent those of their affiliated organizations, or those of the publisher, the editors and the reviewers. Any product that may be evaluated in this article, or claim that may be made by its manufacturer, is not guaranteed or endorsed by the publisher.
